# Integrating deep learning with microfluidics for biophysical classification of sickle red blood cells adhered to laminin

**DOI:** 10.1371/journal.pcbi.1008946

**Published:** 2021-11-29

**Authors:** Niksa Praljak, Shamreen Iram, Utku Goreke, Gundeep Singh, Ailis Hill, Umut A. Gurkan, Michael Hinczewski

**Affiliations:** 1 Department of Physics, Case Western Reserve University, Cleveland, Ohio, United States of America; 2 Department of Physics, Cleveland State University, Cleveland, Ohio, United States of America; 3 Department of Mechanical and Aerospace Engineering, Case Western Reserve University, Cleveland, Ohio, United States of America; 4 Department of Biomedical Engineering, Case Western Reserve University, Cleveland, Ohio, United States of America; University of Khartoum, SUDAN

## Abstract

Sickle cell disease, a genetic disorder affecting a sizeable global demographic, manifests in sickle red blood cells (sRBCs) with altered shape and biomechanics. sRBCs show heightened adhesive interactions with inflamed endothelium, triggering painful vascular occlusion events. Numerous studies employ microfluidic-assay-based monitoring tools to quantify characteristics of adhered sRBCs from high resolution channel images. The current image analysis workflow relies on detailed morphological characterization and cell counting by a specially trained worker. This is time and labor intensive, and prone to user bias artifacts. Here we establish a morphology based classification scheme to identify two naturally arising sRBC subpopulations—deformable and non-deformable sRBCs—utilizing novel visual markers that link to underlying cell biomechanical properties and hold promise for clinically relevant insights. We then set up a standardized, reproducible, and fully automated image analysis workflow designed to carry out this classification. This relies on a two part deep neural network architecture that works in tandem for segmentation of channel images and classification of adhered cells into subtypes. Network training utilized an extensive data set of images generated by the SCD BioChip, a microfluidic assay which injects clinical whole blood samples into protein-functionalized microchannels, mimicking physiological conditions in the microvasculature. Here we carried out the assay with the sub-endothelial protein laminin. The machine learning approach segmented the resulting channel images with 99.1±0.3% mean IoU on the validation set across 5 *k*-folds, classified detected sRBCs with 96.0±0.3% mean accuracy on the validation set across 5 *k*-folds, and matched trained personnel in overall characterization of whole channel images with *R*^2^ = 0.992, 0.987 and 0.834 for total, deformable and non-deformable sRBC counts respectively. Average analysis time per channel image was also improved by two orders of magnitude (∼ 2 minutes vs ∼ 2-3 hours) over manual characterization. Finally, the network results show an order of magnitude less variance in counts on repeat trials than humans. This kind of standardization is a prerequisite for the viability of any diagnostic technology, making our system suitable for affordable and high throughput disease monitoring.

## Author summary

Among the most serious consequences of sickle cell disease are enhanced adhesive intereactions between sickle red blood cells and the walls of blood vessels, leading to potentially fatal vaso-occlusive crises. To better understand these interactions, microfluidic experiments inject patient blood into cavities lined with proteins that tether the red blood cells. The numbers and shapes of the adhered cells provide important clues about the underlying biophysics of the disorder, and could allow detailed patient-specific monitoring of the disease progression. However a major bottleneck in moving this technology from the lab to the clinic has been reliance on specially trained workers to manually count and classify cells, which can take hours for each experimental image. Using deep learning image analysis, we demonstrate a way to address this problem: an automated system for biophysical characterization of adhered sickle red blood cells in microfluidic devices. Our system can achieve accuracies that not only match those of human experts, but are more consistent, showing less variance in counts on repeat trials. Moreover the processing takes minutes rather than hours per image. Our results are a first step toward enabling fast and affordable next-generation microfluidic monitoring tools for sickle cell disease.

## 1 Introduction

### 1.1 Background

Sickle cell disease (SCD) affects over 100,000 Americans and more than 4 million genetically predisposed individuals worldwide [1–4]. The affected demographic commonly draws on ancestral lineage from parts of Africa and India. The most common form of SCD is caused by a single mutation in the *β* globin gene, leading to the expression of an abnormal form of hemoglobin, HbS, in red blood cells (RBCs). Although SCD originates from a single deficit gene, there are many observed clinical sub-phenotypes associated with the disease. They are not mutually exclusive and some of the associated complications are seen to cluster together, suggesting independent genetic modifiers as their epidemiological underpinnings [1]. These sub-phenotypes are associated with different acute and/or chronic complications. Common acute complications include pain crises, acute chest syndrome, stroke and hepatic or splenic sequestration. More long term effects include chronic organ damage of the lungs, bones, heart, kidneys, brain, and reproductive organs [5]. The resultant heterogeneity among SCD patients belonging to different disease sub-phenotypes underlies the need for new methodologies to allow intensive patient specific evaluation and management in outpatient, inpatient and emergency department settings [6]. SCD also requires early diagnosis after birth and constant clinical monitoring through the life-span of the patient, the absence of which leaves them prone to reduced quality of life and premature mortality [7, 8].

The underlying biophysics of SCD hinges on associated complex dynamical phenomena playing out in the vascular flow environment. Mutated hemoglobin molecules expressed in affected sickle RBCs (sRBCs) have a tendency to polymerize in oxygen starved environments, forming long chains which distort the cell profile. The damaged cell membrane displays morphological sickling (distortion into a crescent shape) which dislocates the membrane molecules and leads to a stiffer membrane scaffolding. Consequently sRBCs are more adhesive and less deformable than healthy RBCs [9, 10]. This increased membrane rigidity, along with altered adhesion characteristics that heighten interactions with the endothelium and plasma, directly give rise to SCD’s key manifestation: recurring, painful vaso-occlusive crisis events triggered by sRBC aggregation and blood vessel clogging [4, 11, 12]. The problem thus lends itself very naturally towards exploration in a microfluidic or adhesion assay setup. An important line of investigation in such studies is the search for predictive indicators of disease severity in terms of biophysical rather than molecular markers [8, 13–15]. Microfluidic platforms used for evaluation of sRBC adhesion dynamics have the advantage of being able to directly use clinical whole blood taken from SCD patients [8, 11, 16–18]. This is a versatile laboratory setup that allows one to mimic the complex vascular environment, and realistically explore the multiple, interconnected factors at play. These devices are thus good candidate tools for batch quantitative analyses of the mechanisms occurring in micro-vasculature prior to and during crises, as well as for testing intervention mechanisms [8].

In this study, we focus on one particular microfluidic platform, the SCD Biochip [16, 19]—a customizable, in-vitro adhesion assay where the microchannels can be functionalized with various endothelial or sub-endothelial proteins, and integrated with a programmable syringe pump unit that can implement physiologically relevant flow conditions. The analysis of the data from clinical whole blood samples injected into the SCD Biochip and similar experimental approaches has been challenging, with a major bottleneck being manual counting and categorization of cells from complex phase contrast or bright field microscopic images. Manual quantification of these images is a rigorous, time consuming process and inherently reliant on skilled personnel. This makes it unsuitable for high throughput, operationally lightweight, easily replicable studies. For example, manual cell counting and classification into morphology based sub-groups using the SCD Biochip platform tends to take upwards of 3 hours per image for trained experts. The need for a reliable, fully automated image segmentation, classification, and analysis scheme is thus paramount.

Here we present a standardized and reproducible image analysis workflow that eliminates the need for user input and is capable of handling large amounts of data, by utilizing a machine-learning-based framework that analyzes SCD BioChip assay images in a matter of minutes. Several earlier studies have explored machine and deep learning approaches for automating SCD image analysis [20–23] on RBC microscopy data for other pertinent segmentation and classification problems—like distinguishing healthy from sickle RBCs [21–23], or quantifying shape factor metrics for identified sRBCs [20]. These studies illustrated the power of deep learning to distinguish morphological details at different life stages of the cell, or identify sRBCs en masse from whole blood smears. Building on this progress, the main contributions of our current study are as follows: i) We use deep learning for the first time to analyze morphological details of sRBCs in a context that closely mimics the micro-vasculature in vivo, with cells from whole blood adhering to proteins under flow conditions. This involves solving a two-step problem: first distinguishing sRBCs from other objects in the channel images, and then classifying them into sRBC sub-types. ii) We correlate the morphological differences used in the sub-type classification to biomechanical properties of the cells, which in turn are related to the degrees of HbS polymerization and sickling that characterizes SCD disease progression. These two aspects—the automated analysis of sRBCs adhered to proteins in flow conditions and connecting the morphology of adhered cells to the underlying biomechanics—are the key innovations of our approach.

Our processing pipeline has been set up to be of use as a high throughput tool with detection, tracking, and counting capabilities that could be harnessed to assess visual bio-markers of disease severity. In the long term, this makes our workflow highly suitable for integration into comprehensive monitoring and diagnostic platforms designed for patient specific clinical interventions—a key component of emerging potentially curative therapies like allogenic hematopoietic stem cell transplantation (HSCT) and targeted gene therapies [24–26].

### 1.2 Complexity of classification in whole blood imaging

While significant progress has been made in understanding SCD pathogenesis [4, 27], full characterization of the complex interplay of factors behind occlusion events, and designing appropriate advanced therapeutic interventions, remain significantly challenging. Part of the challenge lies in recreating the conditions of the complex vascular environment, which is the overall goal of the SCD BioChip around which our workflow is designed. Along with the ability to control aspects like applied shear forces and choice of channel proteins, the microfluidic BioChips work with clinical whole blood samples. This lets the experimental setup approximate in vivo conditions as closely as possible, at the cost of significantly increasing the complexity of the image processing problem. Here we describe the various categories of objects—of both cellular and extra-cellular origin—that show up in our channel images. The segmentation process must thus be able to not only identify the sRBCS, but also distinguish them from these other objects with a reliable degree of accuracy.

**Fig 1:**
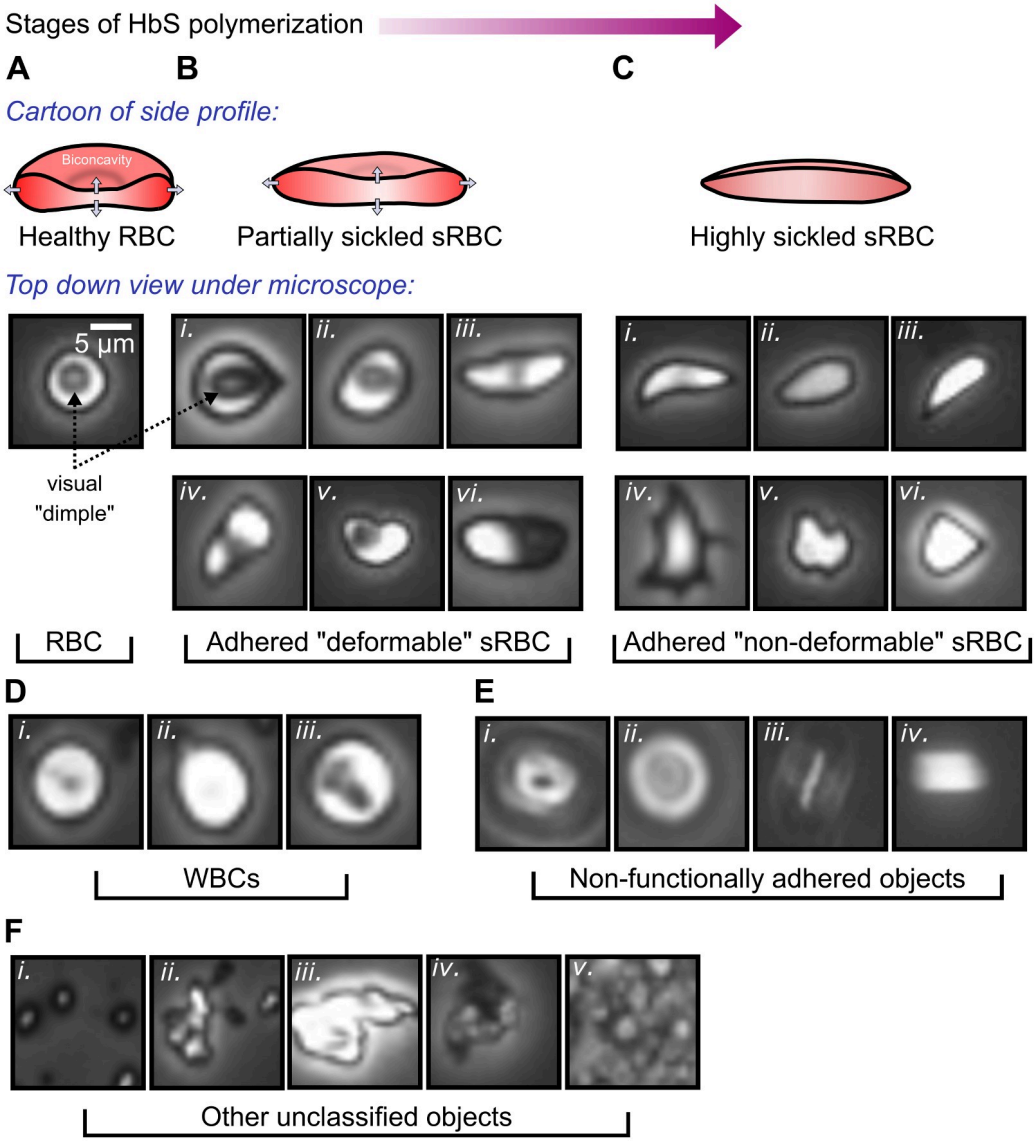
Object categories in our images. **(A-C)** The SCD pathogenetic pathway and changes undergone by the diseased RBC. **A**: A healthy RBC with biconcavity. The latter appears as a dimple viewed from the top. **B (i-iii)**: Partially sickled sRBCs at increasing stages of sickling. The bi-concavity distends out to give a shallower dimple, and elongation in profile. This is the category we identify as *deformable* sRBC (see Section 1.3). **B (iv-vi)**: Additional representative image variants of this category. **C (i-iii)**: Highly sickled sRBCs. The dimple has completely disappeared and the shape is highly elongated. We classify these into our *non-deformable* category. **C (iv-vi)**: More variants in the non-deformable category. Factors like local flow patterns, applied shear forces, and oxygen levels in the environment give rise to various shapes (teardrop, star-like, amorphous) for different sRBCs. **D**: White blood cells (WBCs). **E**: Non-functionally adhered objects. **F**: Other unclassified objects, like **(i)** platelet clusters, **(ii-iii)** lysed cells, **(iv-v)** dirt and dust. In our workflow types **D-F** are classified together in the *non-sRBC* category.

- **RBCs**: Healthy RBCs are easily identifiable from their circular shape with an apparent dimple arising from a top-down view of the bi-concave cell profile (Fig 1A). Since the channels are functionalized with proteins showing preferential adherence for sRBCs, very few healthy RBCs show up in our images.
- **Adhered sRBCs**: SCD pathogenesis (progressive stages of HbS polymerization) causes diseased RBCs to undergo deformation of their cell profile, going from a round to a more elongated, spindle-like shape. Simultaneously, the bi-concavity starts distending outwards. Examples of such partially sickled cells are shown in Fig 1B. Cells at a stage of advanced disease progression, accelerated in hypoxic environments, become highly needle-like in shape, and completely lose their concavity. Examples of such highly sickled cases are shown in Fig 1C. These two categories of adhered sRBC also correlate with biomechanical characteristics of the cell membrane, and we will label them by their membrane deformability, as described in more detail in Section 1.3: *deformable* (partially sickled) and *non-deformable* (highly sickled) sRBCs.
- **White blood cells (WBCs)**: Laminin, our choice of functionalization protein for this study, has known sRBC binding capabilities, and shows little WBC adhesion. Thus our channel images exhibit WBCs with far less frequency relative to sRBCs. The WBCs can be identified from a regular, round shape and smooth appearance, with varying degrees of internal detail (Fig 1D).
- **Non-functionally adhered objects**: The focal plane of the microscope objective in the experiments is set to the protein-functionalized bottom of the channel. Objects adhered to this surface are thus in focus. Due to the finite height of the channel, non-specifically adhered objects outside the focal plane—stuck to the PMMA coverslip on the channel (Fig 1Ei–1Eiii) or flowing by in motion (Fig 1Eiv)—show up as out-of-focus objects. They exhibit characteristic diffraction rings or a blurred appearance.
- **Other unclassified objects**: Various categories of other objects can also appear in the images. Examples include platelet clusters (Fig 1Fi), cellular debris from lysed cells (Fig 1Fii and 1Fiii), and dirt/dust (Fig 1Fiv and 1Fv).

Along with these objects, the background itself can show considerable variation in luminosity and level of detail, depending on the sample and experimenter. A useful processing workflow should be able to deal with these challenges as well.

### 1.3 Establishing the biophysical basis for our classification problem

The observed range of heterogeneities in SCD clinical sub-phenotypes, stemming from the same monogenic underlying cause, remain ill understood [1, 28]. This lack of understanding sets the basis for the specific target problem that motivated our deep learning workflow. In a 2014 study from our group, Alapan *et al*. observed heterogeneity within sRBCs in terms of deformability and adhesion strength [11]—two biophysical characteristics that are key hallmarks of SCD pathogenesis. This observation revealed two new sub-classes with distinct morphologies, called deformable and non-deformable sRBCs (see Fig 1B and 1C). The cells corresponding to the deformable class retain the RBC bi-concave detail, while the non-deformable sRBCs completely lose the bi-concave feature. This bi-concave feature for deformable sRBCs is visible to the naked eye in most ideal cases (see Fig 1Bi, 1Bii and 1Bv). However, based on the variety of configurations of the cell while adhered to the microchannel wall (see Fig 1Biv and 1Bvi), in many cases detecting deformable sRBCs via human analysis can be complicated and inconsistent. These difficulties underline the importance of implementing deep learning models to quickly and consistently count and classify adhered cells.

As their name suggests, deformable sRBCs have relatively flexible shapes that can easily deform under a variety of physiologically relevant flow conditions. However, as RBCs fully progress through the sickling process, not only do the cells ultimately lose the concave dimple feature, but they also become stiffer. These so-called non-deformable sRBCs are readily distinguishable from deformable RBCs based on their sharper edges along with their missing dimples (see Fig 1C). Below in Results Section 3.1 we demonstrate that these morphological differences correlate to significantly altered biomechanical properties in our experimental setup. Furthermore, in addition to their deformability characteristics, these two types of cells are also distinguishable in terms of their adhesion strength to endothelial and sub-endothelial proteins under fluid forces, making them potentially significant for understanding the biophysics of vaso-occlusive crises. In subsequent experiments that integrated a micro-gas exchanger with microfluidics, SCD heterogeneity is more dramatic under hypoxic conditions [29], a known precursor for the onset of these crises.

A wealth of information can be extracted by studying the morphological heterogeneity of sRBCs as a predictive indicator relevant to SCD pathogenesis and adhesion dynamics. Thus our automated deep learning workflow focuses on the above described SCD heterogeneity: counting sRBCs adhered to proteins in our microfluidic setup, and classifying these adhered cells into deformable and non-deformable types. Because the input consists of complex microscopy images of whole blood, the approach has to reliably disregard non-adhered sRBCs (see Fig 1E) and other miscellaneous objects (see Fig 1F).

## 2 Materials and methods

### 2.1 Details of the experimental assay, image collection, computational hardware and software

RBC adhesion was measured using an in vitro microfluidic platform developed by our group, the SCD Biochip [16, 19]. The SCD Biochip is fabricated by lamination of a polymethylmethacrylate (PMMA) plate, custom laser-cut double-sided adhesive film which has a thickness of 50 *μ*m (3M, Two Harbors, MN) and an UltraStick adhesion glass slide (VWR, Radnor, PA). The width and the length of the channel are 4 mm and 25 mm, respectively. The glass slide is treated with GMBS (N-*γ*-maleimidobutyryl-oxysuccinimide ester) to facilitate chemisorption of the functional protein laminin. Laminin is a sub-endothelial protein with preferential adherence to sRBCs over healthy RBCs [30], allowing us to focus on sRBC characterization. 15 *μ*l of whole blood collected from patients diagnosed with SCD at University Hospitals, Cleveland, Ohio, was perfused into the microchannels functionalized with laminin (Sigma-Aldrich, St. Louis, MO).

Images for the deep learning analysis were collected using the following protocol. Shear stress was kept at 0.1 Pa, mimicking the average physiological levels in post-capillary venules. A constant displacement syringe pump was used for injecting blood samples at a constant flow rate of 1.85 *μ*l/min, which is calculated based on the average blood viscosity of SCD samples as reported in an earlier study from our group [31]. After the non-adherent cells were removed by rinsing the microchannels with 0.2% w/v bovine serum albumin containing saline buffer at the same shear stress as blood, microfluidic images were taken by an Olympus IX83 inverted motorized microscope. Mosaic images in phase-contrast mode with an integration time of 0.5 ms were recorded and then stitched together by Olympus CellSense live-cell imaging and analysis software coupled with an QImaging ExiBlue Fluorescense Camera. An Olympus 10x/0.25 long working distance objective lens was used for imaging. For the separate deformability analysis of Results Section 3.1, channel images were first captured under constant flow conditions of 10 *μ*L/min (which corresponds to a shear rate of about 100/s), and then subsequently captured again after the flow was turned off.

In terms of our computational hardware, we ran and trained our neural networks on an NVIDIA GeForce RTX 2080Ti GPU, with a Intel Core i7–8700K 3.7 GHz 6-Core CPU, and 32 GBs of RAM. All codes are developed in Python 3 making use of the scikit-learn [32], NumPy [33], Keras [34], and Tensorflow [35] libraries. Jupyter Notesbooks implementing our methods are hosted on GitHub (https://github.com/hincz-lab/DeepLearning-SCDBiochip).

### 2.2 Overview of the image analysis workflow

We designed a bipartite network consisting of two individually trained neural networks that work in tandem to quantify our whole channel microfluidic image data. The workflow contains two phases of analysis that involve convolutional neural nets for cell segmentation/detection and classification of adhered sRBCs. We found this bipartite approach helpful in streamlining our workflow, trimming unnecessary operational bulk, and significantly improving performance metrics.

**Fig 2:**
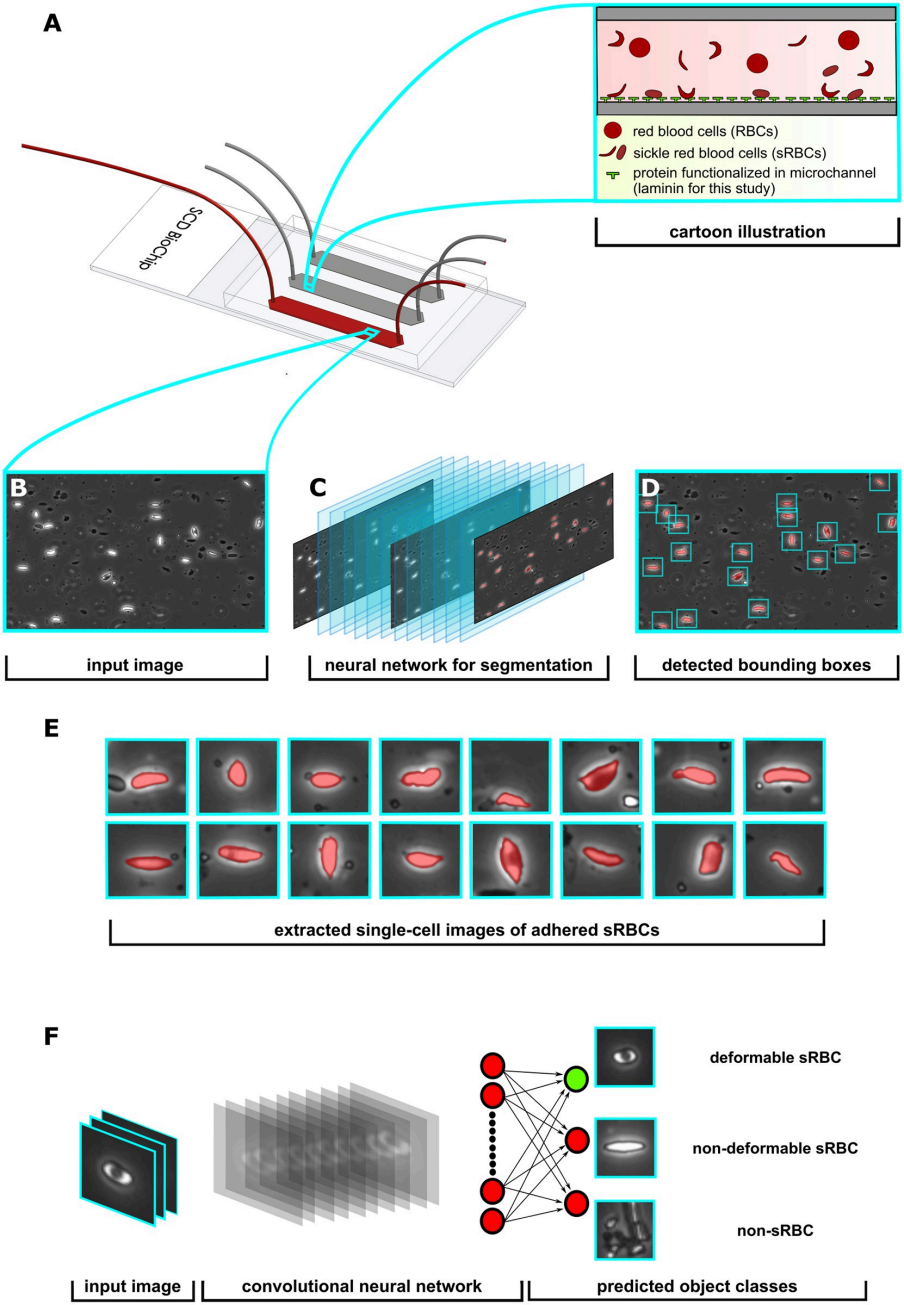
Overview of processing pipeline. **A**: SCD BioChip and cartoon illustration represents an in-vitro adhesion assay and adhesive dynamics of sRBCs within a mimicked microvasculature. **B**: Generated input image fed into the Phase I network. **C**: Phase I segmentation network predicts pixels belonging to adhered sRBCs, shaded red in the images. **D**: Drawing bounding boxes around segmented objects. **E**: Extracting adhered objects into individual images. **F**: The input layer of the Phase II classifier network receives an image from the Phase I detection network, then performs a series of convolutions and nonlinear activations to finally output class predictions.

A schematic of the processing pipeline described here is shown in Fig 2. Each phase of the pipeline has been built around a separate neural network. Since we are dealing with vast amounts of complex microscopy data that contains a plethora of cellular objects under fluid flow, we created Phase I to deal with object detection of adhered sRBCs exclusively. For Phase II (Fig 2F) we focused on the biophysical classification of sRBCs into deformable and non-deformable types. After collecting microchannel images from the SCD BioChip, the workflow first implements Phase I, which consists of a convolutional neural net with an architecture that downsamples and then upsamples the input image data into a segmentation mask (Fig 2A–2C). The downsampling portion of the network constructs and learns feature vectors as input data for the upsampling part of the neural network, allowing it to find segmentation masks for the original input images [36]. After each convolution layer before a downsampling or upsampling operation, we apply a batch normalization layer, improving training time and acting as a regularizer [37]. With each layer implementation, these batch normalizations introduce two learnable parameters for normalizing the corresponding intermediate features. We also include copy and concatenations which incorporate skip connections from the encoder to the decoder layers. These operations have been motivated by the success of U-Net, a semantic segmentation network heavily used in the biomedical image segmentation community [38].

Given an input image, the network learns to assign individual pixels to three categories: background, adhered sRBC, and non-functionally adhered / other. The non-functionally adhered / other category largely involves detached or freely flowing cells (i.e. cells not attached to proteins along the channel wall, as seen in Fig 1E), which are easily distinguishable from adhered cells. We trained our encoder-decoder model using a loss function that combines the cross-entropy loss $L_{CE}$ and Jaccard loss $L_{Jac}$. The $L_{CE}$ penalizes individual per-pixel segmentation, while the $L_{Jac}$ penalizes the network based on the intersection over the union between the predicted and ground truth segmentation mask. The latter is useful for training networks on segmentation tasks with imbalanced pixel classes.

The three-class pixel labeling scheme we have used for our Phase I segmentation network is a first step toward capturing the complexity of features in our images. Ultimately however we are interested in classifying entire cell objects as either deformable sRBC or non-deformable sRBC rather than individual pixels corresponding to adhered cells. Thus further refinement is necessary, motivating the introduction of our Phase II network. We can rely on the Phase I network to accurately identify clusters of pixels as adhered sRBCs. The algorithm then computes 32 × 32 pixel bounding boxes around such clusters, each box centered around the cluster centroid (Fig 2D). The size of the box takes into account the average size of the sRBCs in our channel images at 10x magnification, so that one box typically contains an entire cell. These boxes then form a set of images (Fig 2E) that are the input for our Phase II network.

In Phase II (Section 2.4), the images are run through a convolutional neural net for biophysical classification. The neural network performs a series of convolutions and filtering operations, which ultimately classifies the image as a deformable sRBC, non-deformable sRBC or non-sRBC (Fig 2F). If the Phase I analysis was entirely error-free, there would of course be no input images in Phase II corresponding to non-sRBC objects. But we include this category to filter out the rare mistakes made by the Phase I analysis, further enhancing the accuracy of the results at the completion of Phase II. Our dataset for Phase II consisted of a library of 6,863 manually classified cell images. Since this is a modestly-sized dataset for training an image classification network, we decided to implement transfer learning, an approach that can enhance training in cases with limited data [39]. We found that the deep residual network called ResNet-50 [40], pretrained on ImageNet [41], worked well in learning morphological features for our biophysical classification task. Since the bottom and middle layers of neural networks contain mostly general feature representations, which are then applicable to many datasets and tasks, we can achieve a performance boost in our models by transferring model weights trained on the ImageNet task. Even though our sRBC classification task is not the same as the ImageNet task, we see benefits in terms of performance and training time [42, 43]. This is consistent with earlier work, where it has been showed that transferring weights from a model trained on ImageNet can boost performance on a biomedical image task [44]. We also conducted a *k*-fold cross-validation protocol to estimate the accuracy of our machine learning model on validation data taken from images not included in the network training, including whole channel images from separate experiments. The details of the architectural design, data set pre-processing and preparation, progress checkpoints, and evaluation metrics for each network phase are presented in the next two sections.

### 2.3 Phase I: Detecting adhered sRBCs

This section is ordered as follows. First, we present the details of our neural network for semantic segmentation with an architecture inspired by encoder-decoder models [36, 38]. We describe our preprocessing procedure for expanding our training set with augmentation data to overcome issues with imbalanced pixel classes during training. Finally, we illustrate overall performance of the network in detecting adhered sRBC cells by presenting multiple relevant evaluation metrics: pixel accuracy, intersection over union (IoU), and the Dice coefficient.

#### Preprocessing of microchannel images and preparation of the data set

Before we implement the neural network for segmentation and detection, we record mosaic images of a single whole channel and stitch each image together, leading to a larger image with pixel dimensions 15,000 × 5,250. We then split the raw whole channel image into 3,500 equally-sized tiles by dividing the rectangular image with 100 vertical and 35 horizontal partitions, leading to tiles with pixel dimensions 150 × 150.

For our optimal architecture, the network has an input layer with size 128 × 128 × 3, with the first two dimensions representing height and width in pixels, and the last dimension representing three channels. Though our tile images were all grayscale, their format varied depending on the experimental procedure for recording the photo, with some having three channels and some just one channel. In the latter case we copy the first channel and then concatenate the copied channel two more times, creating images with three-channel depth. We then resize the width and height of the tile from 150 × 150 × 3 to 128 × 128 × 3 with a bicubic interpolation function, to match the input specifications of the network, and apply zero-centered normalization. See Table 1 for a summary of the data set details.

**Table 1:** Details of data sets used for training / validating the neural networks in the two phases of our workflow. For both Phase I and II, we use k-fold cross validation with *k* = 5, and split the respective data sets so that the training and validation sets correspond to approximately 80% and 20% of the whole dataset for each fold.

Phase	Data Set
I	3,500 pixel-labeled tiles (each 128 × 128 pixels)
II	6,863 single-cell images (each 32 × 32 pixels) representing: 3,362 deformable sRBC, 1,449 non-deformable sRBC, 2,052 non-sRBC

Since we are using supervised learning, we require that the data set be manually labeled beforehand. This was accomplished using the Image Labeler app in Matlab R2019a. As described above, each pixel is assigned to one of three labels: background (0), adhered sRBC (1), and non-functionally adhered / other (2). The segmentation masks with numerical labels were then converted to one-hot encoded representations.

#### Overcoming imbalanced data for segmentation tasks via augmentation

A common challenge in training semantic segmentation models is class imbalanced data [45]. A class imbalance occurs when the frequency of occurrence of one or more classes characterizing the data set differs significantly in representation, usually by several orders of magnitude, from instances of the other classes. This problem results in poor network performance in labeling the minority classes, a significant challenge for biomedical image segmentation in which frequently the minority class is the one under focus. A typical example is in pathologies such as inflammatory tissues or cancer lesions, where the aberrant tissue patch or lesion is much smaller in size compared to the whole image. This issue leads to reduced capacity for learning features that correlate to the lesions. For our microchannel images, the background far outstrips the adhered sRBCs in representation, heavily skewing the data set. In the absence of balancing, we find the network significantly misclassifies adhered sRBC pixels, in some cases completely ignoring them. Since our interest lies in accurately identifying the adhered cells, it is imperative to address this imbalance and improve accuracy for these minority classes.

As a first step, we located image tiles within our initial dataset corpus that contained at least one annotated adhered cell or non-functionally adhered cell / other. This procedure led to finding a total of 877 images with at least one adhered cell and 360 images with at least one non-functionally adhered / other cell. From here, we augmented these tiles by 90, 180, and 270 degrees, leading to a total of 2631 (adhered cell) and 1080 (non-functionally adhered / other) new training dataset samples. Overall, by expanding the original dataset corpus with this augmentation protocol to 7211 unique samples, we were able to help balance the training procedure. In addition, by rotating images we can introduce new cell orientations that are physically relevant features for the model to learn.

Furthermore, to prevent overfitting we introduced additional data augmentations during each epoch of the training. These were applied only to the training subset [46], with the validation subset unaltered. We utilized both random horizontal and vertical reflections for this augmentation process: an image was reflected with a probability of 50% during each iteration. We also augmented the images with random rotations of angles between the values -90 and 90 degrees. With the combination of random reflections and rotations, new cell orientations were introduced. More importantly, since the neural network constantly was presented different features for each epoch, the model had a lower chance of overfitting on the training set.

#### Constructing the Phase I network architecture

Our Phase I network is an encoder-decoder model which implements convolutional blocks that contain filters, nonlinear activation functions (ReLU), batch normalization, down (max), and up (transpose) sampling layers. The final layer is a softmax which predicts a one hot encoded tensor, corresponding to one of the three classes: background, adhered sRBC, and non-functionally adhered / other. For our loss function, we choose to combine the binary cross-entropy $L_{CE}$ and Jaccard loss (i.e. intersection over union loss) $L_{Jac}$, expressed as:

$$\begin{array}{c} L=L_{CE}+L_{Jac}, \end{array}$$ (1)

where

$$\begin{array}{c} \begin{array}{cc} L_{CE} & =-\frac{1}{N}\sum_{\alpha =1}^{N}\sum_{i=1}^{3}(p_{i}^{\left(\alpha \right)}\;\text{log}\left(q_{i}^{\left(\alpha \right)}\right)+\left(1-p_{i}^{\left(\alpha \right)}\right)\;\text{log}\left(1-q_{i}^{\left(\alpha \right)}\right)),\\ L_{Jac} & =-\text{log}(\frac{\sum_{\alpha =1}^{N}\sum_{i=1}^{3}p_{i}^{\left(\alpha \right)}q_{i}^{\left(\alpha \right)}}{2N-\sum_{\alpha =1}^{N}\sum_{i=1}^{3}p_{i}^{\left(\alpha \right)}q_{i}^{\left(\alpha \right)}})\equiv -\text{log}\left(J\right). \end{array} \end{array}$$ (2)

Here *N* represents the number of data points in a batch to be analyzed, $p_{i}^{\left(\alpha \right)}$ the *i*th component of the one hot encoded ground truth probability for the *α*th data point, and $q_{i}^{\left(\alpha \right)}$ the corresponding predicted softmax probability component. $L_{Jac}$ is the negative logarithm of the Jaccard index *J*, whose numerator is a measure of the size of the intersection between the ground truth segmentation mask and the predicted segmentation mask. The denominator is a measure of the size of the union between these two masks. Note that the 2*N* in the denominator represents the total size of the two masks (union plus intersection), so subtracting the intersection (the expression in the numerator) from 2*N* gives the size of the union. Furthermore, we compared our encoder-decoder segmentation model, which is tuned and optimized on our sRBC dataset, against the most recent state-of-the-art segmentation model called HR-net [47], which introduces novel connections between high-to-low resolutions in parallel during training. For both model architectures, the initialized weight parameters were sampled from a random normal distribution. We find that our encoder-decoder model performs on par with HR-net on the validation datasets in terms pixel accuracy, IoU, and Dice coefficient metrics (see Fig A in S1 Text). As described below, however, the encoder-decoder analyzes images almost twice as fast as HR-net, and hence was our preferred segmentation approach. The full details of the Phase I network architectures are shown in https://github.com/hincz-lab/DeepLearning-SCDBiochip.

### 2.4 Phase II: Classification into morphological subtypes

#### Setting up the Phase II network architecture

The structure of our Phase II cell classifier network was adapted from ResNet-50, the very deep residual neural network [40]. Residual neural networks implement skip connections in the hopes of avoiding vanishing gradients. Our implementation of ResNet-50 is pre-trained on the reduced ImageNet ILSVRC database, consisting of over 1 million training images and belonging to 1000 different classes [41].

**Fig 3:**
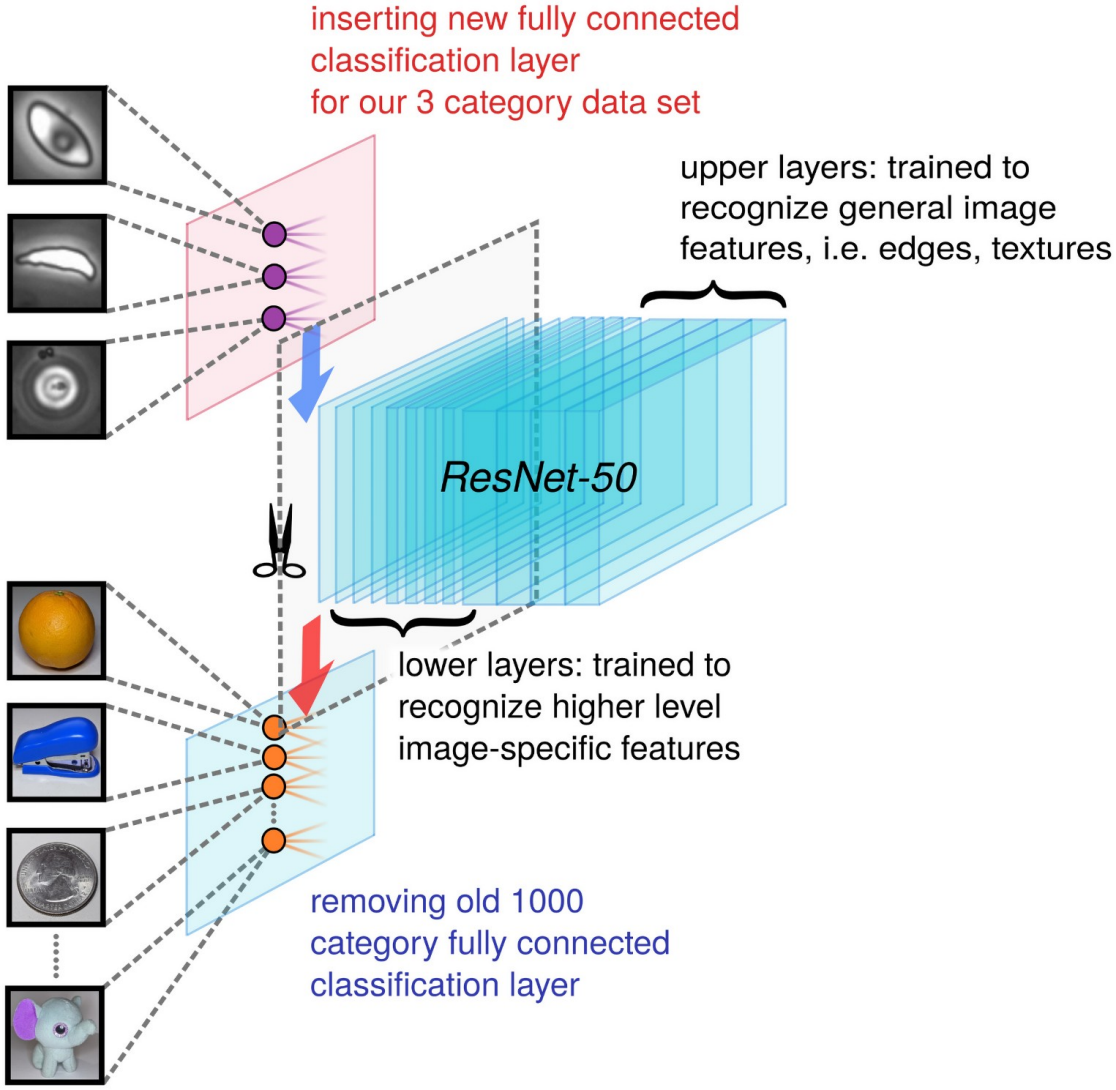
Phase II network. A schematic of the transfer learning workflow used to train our classifier network. We employ the ResNet-50 architecture and start with weights pre-trained on the 1000 category reduced ImageNet ILSRVC database. The final fully connected learnable classification layer is swapped out for a 3 class classification layer suited to our problem.

The overall microfluidic image data set size is still relatively small for our complex classification task, which requires learning subtle morphological features in cells of various sizes, shapes, and orientations. We thus choose to utilize a transfer learning framework [39]: rather than initializing the network with randomly chosen weights, we start with weights pre-trained on ImageNet, allowing us to achieve a higher starting accuracy on our own data set, faster convergence, and better asymptotic accuracy. To tailor the network for our purposes, we added a global 2D average pooling operation, a fully connected layer with a width corresponding to the previous number of feature maps before applying a pooling layer, and swapped out the final fully-connected layer of original ResNet-50 model; in particular, we swapped out the original 1000 neuron output layer with a layer of three output neurons corresponding to the three object classes (Fig 3). As described below, we also checked our results against two other possible choices for the Phase II network: a vanilla convolutional neural network without pre-training, and a pre-trained network based on Xception [48] rather than ResNet-50. Xception implements depthwise separable convolutions, in contrast to ResNet-50 which uses skip connections. More details on all these architectures can be found in the Fig B in S1 Text.

#### Data set preparation for the Phase II classifier

As mentioned earlier, the input images for Phase II are 32 × 32 pixel images corresponding to single cells (see Table 1 for data set details). Ideally these are all adhered sRBCs, but there is a tiny subset of non-sRBC objects, a source of error that the Phase II network is designed to mitigate. The details of constructing these single-cell images are as follows. Starting with the three-class segmentation mask generated at the end of Phase I, we binarize the pixels in these images according to our adhered sRBC pixel class by assigning 1 to sRBC pixels and 0 to non-sRBC pixels. We delete any small objects that form connected clusters of 1 pixels where the cluster size is smaller than 60. This threshold allows us to remove debris from the images, while being small enough relative to the range of sRBC cell sizes to preserve clusters that are actually sRBCs. We compute the centroids of the remaining clusters, ideally corresponding to sRBC cells, and extract 32 × 32 pixel bounding boxes centered at each cluster centroid (see Fig 2D and 2E). Before we input these extracted cell images into the Phase II neural network for biophysical classification, we resize the image from 32 × 32 × 3 to the corresponding ResNet-50 input layer size of 224 × 224 × 3, and apply zero-centered normalization. Among the models we used for comparison, Xception also takes input images of size 224 × 224 × 3, while for the vanilla network we chose an input layer size corresponding to the original image size 32 × 32 × 3. See Fig B in S1 Text for more details.

The training set for our supervised learning in Phase II consists of 6,863 single-cell images in three object categories: deformable sRBC (3,362 images), non-deformable sRBC (1,449 images), and non-sRBC (2,052 images). In terms of curating our data set, we initially started with a batch of individual objects that were manually extracted from a large set of channel images displaying different luminescence and granularity features that covered the broad spectrum of sample and experimental variance (see Fig 1A). However, after we completed our Phase I network, we expanded the data set to include the single-cell images generated by Phase I, though we manually verified the labels to correct any errors. Our data set also covers different physiological conditions like normoxia and hypoxia, which allows the resulting image processing pipeline to handle data from a wide range of SCD assays.

#### Phase II training details

The data set was split randomly into 80% training and 20% validation subsets, and the network was trained with maximum epoch number 30 and minibatch size 32. Each training session had 6450 iterations, and thus 215 iterations per epoch. Similar to the Phase I training protocol, we utilized data augmentation on the training subset to expand the training data set corpus, allowing us to implement powerful networks (e.g. ResNet50) without overfitting [46]. Once again, we utilized both horizontal and vertical random reflections for the augmentation process: an image was reflected with a probability of 50% during each iteration. We also augmented the images with random rotations of angles between the values -90 and 90 degrees. Since both deformable and non-deformable sRBCs have heterogeneous morphologies and our experimental microfluidic setup introduces various cellular orientations along the plane of the microchannel (see Fig 1B and 1C), the rotations and flips help the neural network anticipate this variety. Lastly, we implemented a zero-centered normalization for each image sample.

## 3 Results and discussion

### 3.1 Cellular deformability analysis

**Fig 4:**
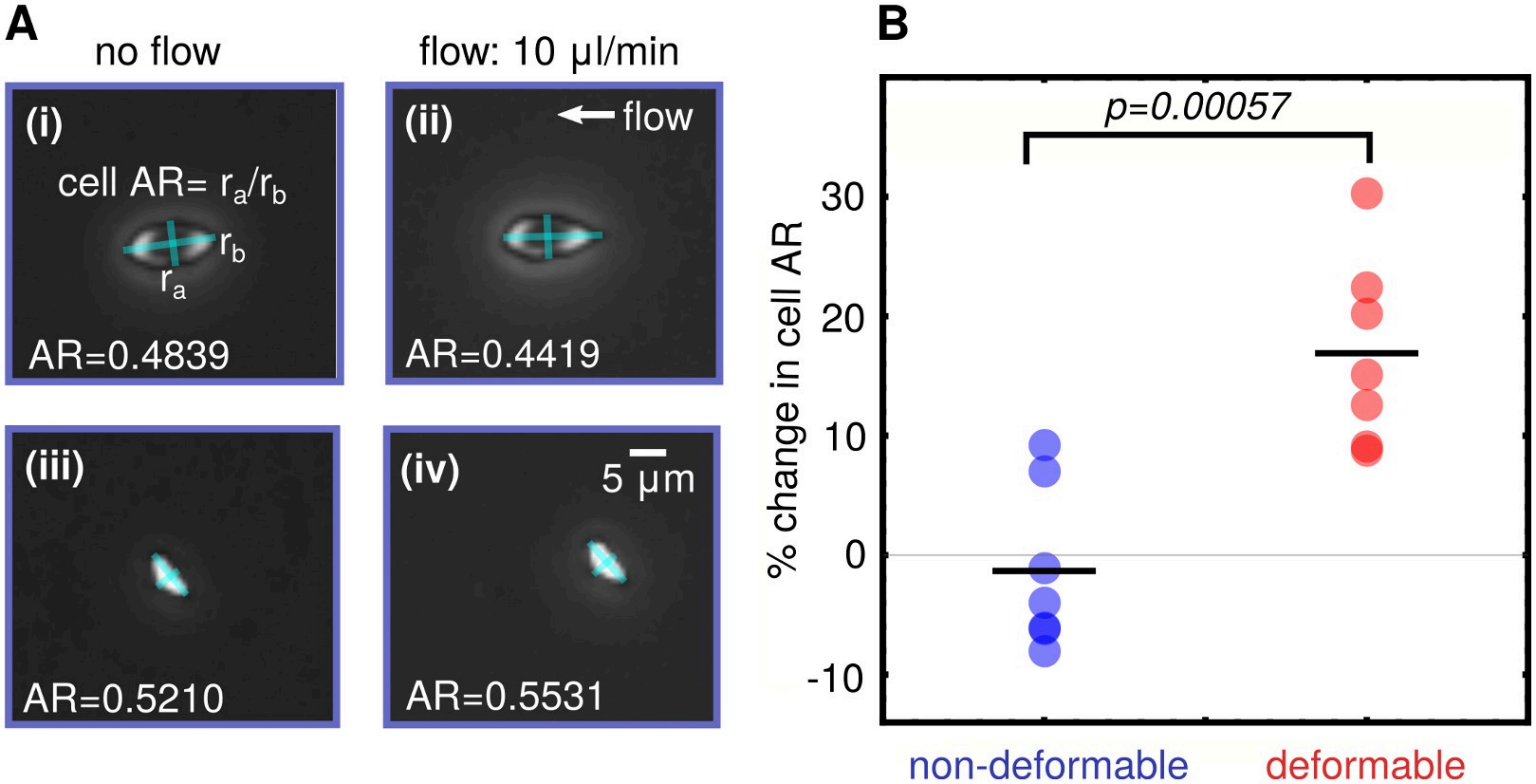
Cell deformability analysis. **A**: Schematic for estimation of change in cell aspect ratio (AR) between flow and no flow conditions. **(i-ii)** show a deformable type cell, and **(iii-iv)** a nondeformable. **B**: Mapping deformability to morphology: Cells visually identified as the deformable morphological subtype show significantly higher percentage change in cell AR between flow and no flow conditions compared to the non-deformable subtype.

To validate the connection between adhered sRBC morphology and deformability in our experimental setup, we analyzed pairs of images of the microfluidic channel first under flow (10 *μ*L/min) and then under no flow conditions. These images were examined to look for sRBCs that had not detached or moved significantly between the two image captures, to allow for legitimate comparison. The relative change in cell aspect ratio (AR) under the two flow conditions was then analyzed for each cell (Fig 4), as a measure of cellular deformability. We have defined the cellular AR as the ratio of the estimated minor to the major axis. A set of 14 cells was identified and manually classified as seven deformable and seven non-deformable according to the morphological characteristics described in Section 1.2. After analyzing the cellular AR of the adhered RBCs under the two flow conditions, we found that the morphology of the sRBCs correlates to the deformability characteristics. The cells classified morphologically as deformable showed a mean change in AR of about 20% on average between flow and no flow. For those classified as non-deformable sRBCs, the average percent change in AR was close to zero. Results are summarized in Fig 4. Given the heterogeneity of the cell shapes and errors introduced by the pixelation of the images, the AR changes of each subtype have a distribution, but the difference in the average AR between the two distributions is statistically significant (*p* = 0.00057). These results reproduce the link between morphology and deformability observed in Alapan *et. al*. [11] in exactly the same experimental setup we use to do the deep learning image analysis. Thus the classification into subtypes produced by the algorithm should be strongly correlated with a key biomechanical feature of the individual sRBCs.

### 3.2 Phase I network performance

#### Segmentation performance evaluation

To quantify overall performance of our Phase I network, we computed two of the most commonly used metrics for semantic segmentation [49]: (i) the Jaccard index *J*, defined in Eq (2), also known as the intersection over union (IoU); (ii) the closely related Dice coefficient *D*, which is the twice the intersection divided by the total number of pixels. It can be expressed in terms of *J* as *D* = 2*J*/(1 + *J*). We have also tracked the pixel accuracy in classifying individual pixels on both training and validation sets.

**Fig 5:**
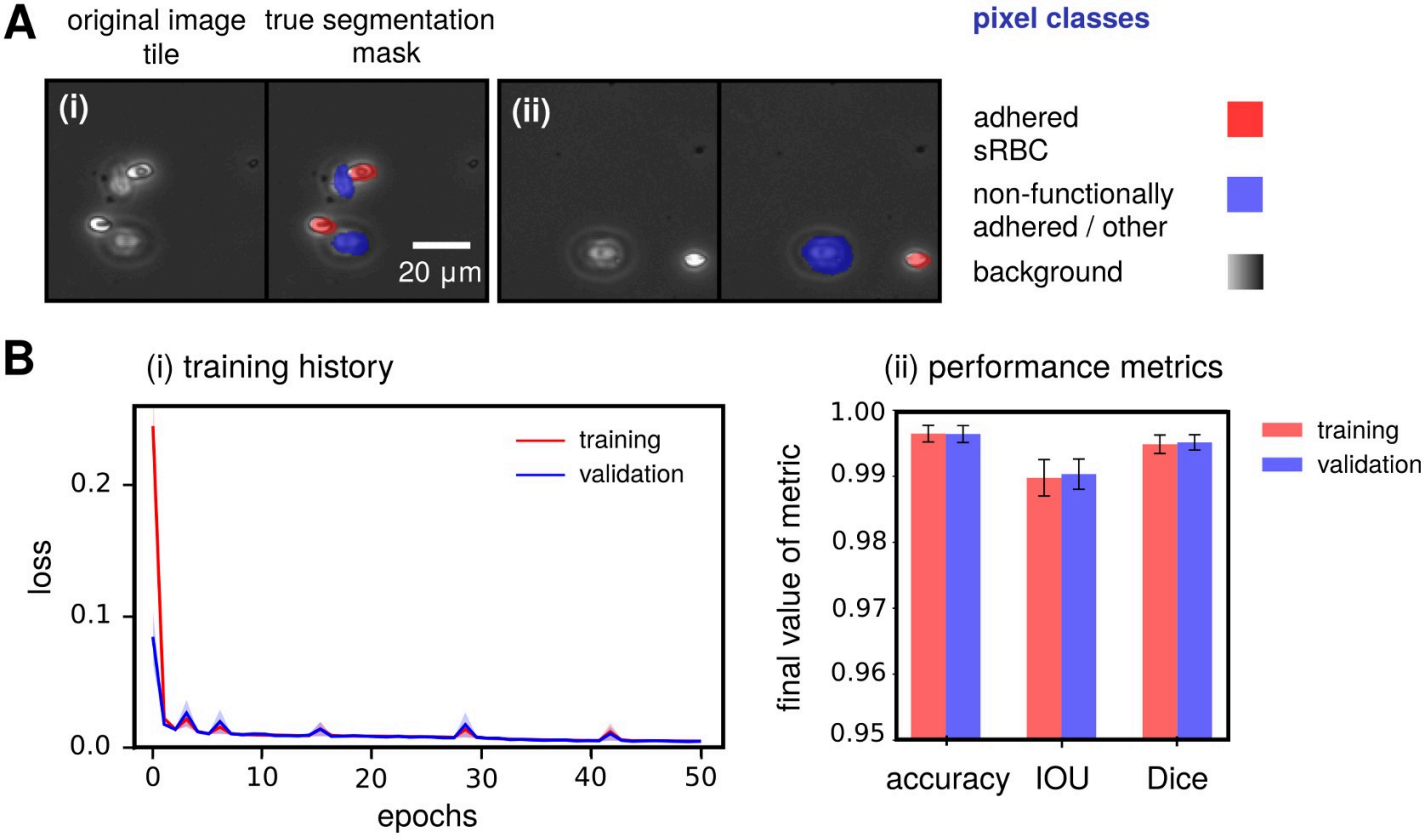
Phase I network performance metrics. **(A)** Two examples of typical input image tiles for the Phase I network, along with the corresponding manually labeled segmentation mask assigning each pixel in the image to one of three pixel classes (listed on the right). **A(i)** shows a tile with deformable sRBCs and non-functionally adhered / other objects, while **A(ii)** shows one with a non-deformable sRBC and other object. **(B)** **(i)** Training and validation history of the total cross entropy / Jaccard loss function L for the Phase I network. The solid curve corresponds to the average loss over 5 folds, while the same colored light band denotes the spread (standard deviation) in the loss over these folds. Training history is shown in red and validation in blue (purple indicates overlap). **(ii)** Final 5-fold averaged performance metric values for both training and validation reached by our Phase I network at the end of training over 50 epochs. Uncertainties indicate spread around the mean of each metric over the 5 folds.

Our Phase I network is successful: it is able to achieve state-of-the-art accuracy in segmentation of channel images from whole blood experiments compared to similar studies in literature [20, 21], reaching 0.997±0.001 pixel accuracy, 0.991±0.003 IoU, and 0.996±0.002 Dice coefficient values on the validation set across all 5 *k*-fold hold-outs (see Fig 5A for example Phase I input tiles and Fig 5B for performance metrics). These metric values are quite similar to the HR-net model [47] trained on the same task, which achieves the following values: 0.997±0.001 pixel accuracy, 0.981±0.006 IoU, and 0.991±0.003 Dice. More details on the training metrics both of these model architectures can be found in Fig A in S1 Text. The main difference between the two models is analysis time: the encoder-decoder takes an average of 72±10 seconds to process one whole channel, compared to 143±8 seconds for HR-net. Given that processing speed is an important consideration in analyzing high-throughput adhesion experiments, we thus preferred the encoder-decoder architecture for segmentation. Care must be taken in interpreting these Phase I metrics, and they should not be naively used as an adequate standalone measure of the overall performance. As is commonly the case, we found that the bulk of our error arose from segmentation of cell boundaries rather than the cell itself. Since we are more concerned about locating centroids of the predicted segmentation masks, to crop and extract sRBC images for classification in Phase II, the cell boundary errors do not significantly affect the final results in our pipeline.

### 3.3 Phase II network performance

To quantify overall performance of our Phase II network, we computed the performance metrics [50] defined below for a given class *i*, where *i* corresponds to either deformable sRBC, non-deformable sRBC, or non-sRBC cell:

$$\begin{array}{c} \begin{array}{cc} \text{Precision}(i) & =\frac{{\text{TP}}_{i}}{{\text{TP}}_{i}+{\text{FP}}_{i}},\\ \text{Recall}(i) & =\frac{{\text{TP}}_{i}}{{\text{TP}}_{i}+{\text{FN}}_{i}},\\ \text{Accuracy}(i) & =\frac{{\text{TP}}_{i}+{\text{TN}}_{i}}{\text{Total}}, \end{array} \end{array}$$ (3)

Here TP_*i*_, TN_*i*_, FP_*i*_, and FN_*i*_ denote the number of true positive, true negative, false positive and false negative outcomes in classifying a given cell image into class *i*. “Total” represents the total number of images involved in the evaluation. Precision indicates the agreement between predicted and target class labels, while recall measures the effectiveness of the neural network’s classification ability when identifying cell classes.

**Fig 6:**
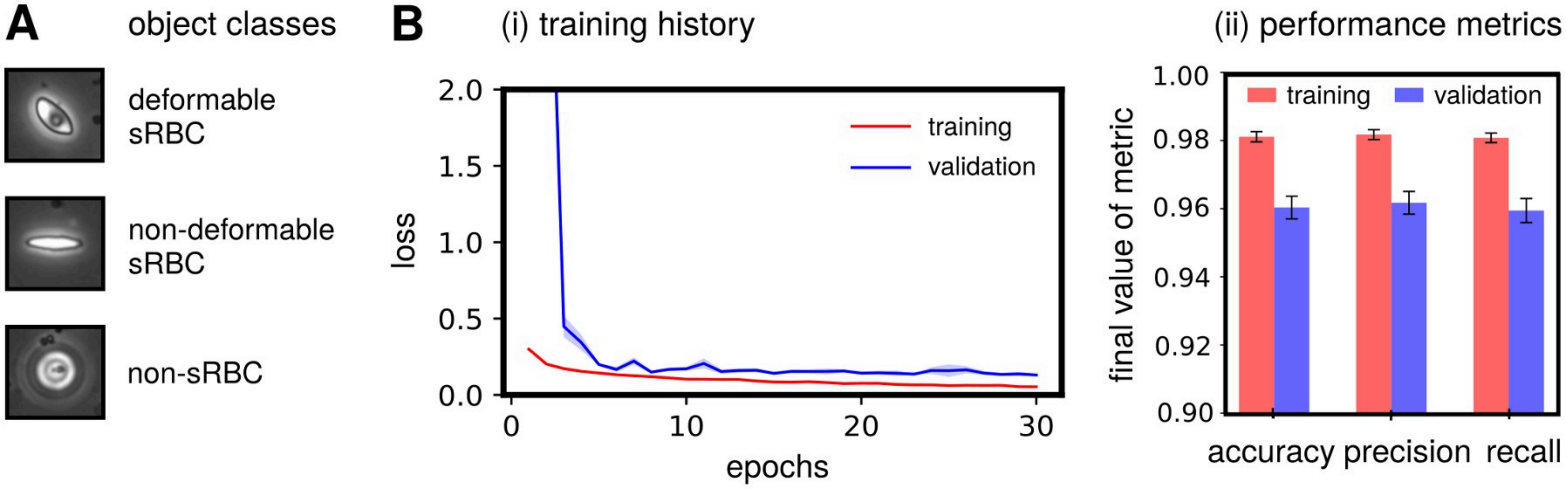
Phase II network performance metrics. **(A)** Representative examples of single-cell images for each classifier category, the input for Phase II. **(B)** **(i)** Training and validation history of the loss function for the Phase II network. The solid curve corresponds to the average loss over 5 folds, while the same colored light band denotes the spread (standard deviation) in the loss over these folds. Training history is shown in red and validation in blue (purple indicates overlap). **(ii)** Final 5-fold averaged performance metric values for both training and validation reached by our Phase II network at the end of training over 30 epochs. Uncertainties indicate spread around the mean of each metric over the 5 folds.

During learning, the network weights are optimized to make the class predictions for the training data set as accurate as possible. However, depending on the training set and the stochastic nature of the optimization process, the accuracy of the network on the testing set can vary. Attention to this issue becomes imperative when dealing with smaller data sets for classification tasks, like in our Phase II case. *k*-fold cross-validation is one approach to validate the overall performance of the network in this scenario. The general procedure starts by shuffling the total data set before splitting it into training/validation subsets, to generate an ensemble of *k* such unique subsets (or folds). We choose *k* = 5, with an 80/20% split for training/validation sets. Each fold consists of a unique combination of 20% of the images as the hold-out (validation) set, and the remaining 80% as the training set. Our combined data set of 6863 total images thus generates five unique folds with training and validation sets containing 5488 and 1372 images each (accounting for rounding off). Finally, we fit the neural network parameters on the training set and evaluate the performance on the validation set for five unique runs. Then for each single run, we collect the training and validation accuracy, listed in Table 2. We also show the mean and standard deviation of all the folds, with the small standard deviation being an indicator that our training did not suffer from overfitting. Fig 6A shows example Phase II input images for each classifier category, while Fig 6B shows metrics highlighting the typical performance, which averaged to 0.960±0.003 accuracy, 0.962±0.003 precision, and 0.959±0.004 recall in object classification over the folds. Furthermore, in terms of loss, accuracy, precision and recall during training, our fine-tuned ResNet-50 model outperforms the vanilla and fine-tuned Xception model variants on the validation set, averaged over the *k*-fold sets (see Table A and Fig C in S1 Text).

**Table 2:** Results from the 5-fold cross-validation of the Phase II network.

Fold No.	Training Accuracy	Validation Accuracy	Training Time
1	0.982	0.957	33 min 43 sec
2	0.978	0.962	33 min 36 sec
3	0.982	0.959	32 min 20 sec
4	0.982	0.957	33 min 21 sec
5	0.981	0.966	33 min 09 sec
Mean	0.981±0.0.002	0.960±0.003	33 min 26 sec

### 3.4 Interpreting Phase II classification results with activation maps

**Fig 7:**
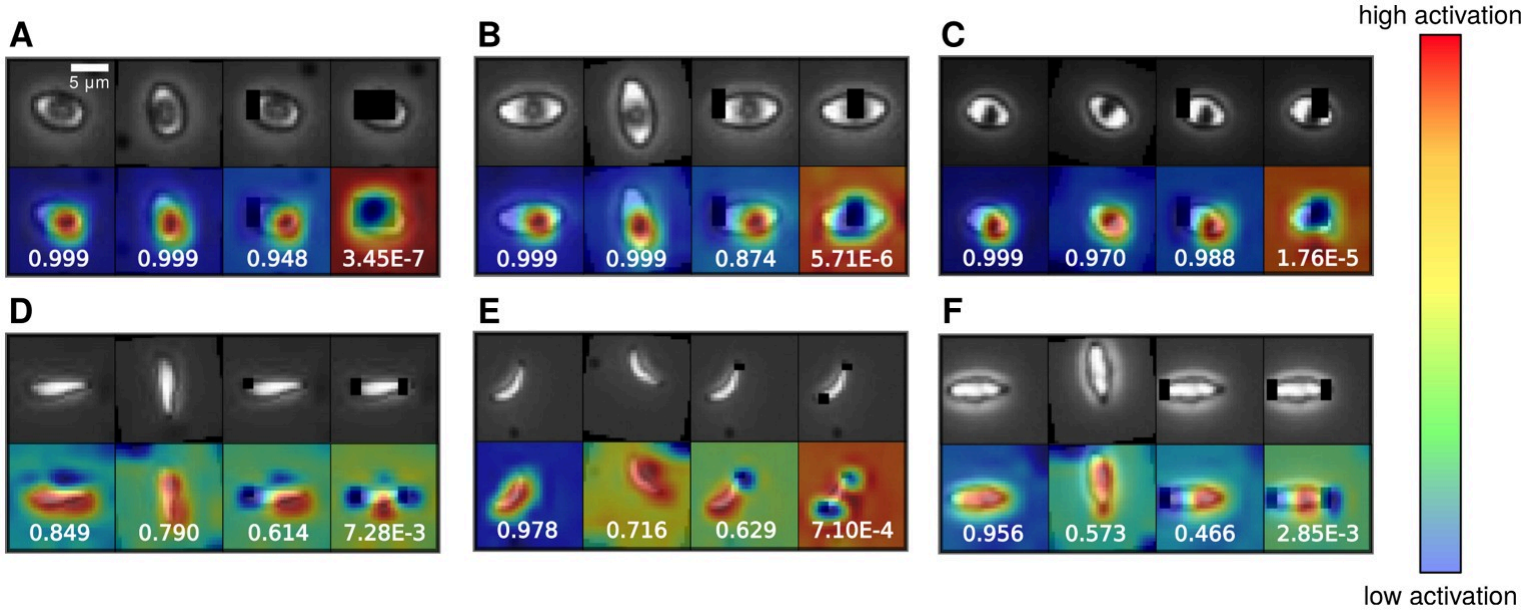
Interpreting the fine-tuned ResNet-50 model. Class activation maps for representative cell types, highlighting the cell features that allow the Phase II network to classify each cell as either deformable or non-deformable sRBC. These heat maps are a measure of the model’s attention [51, 52], where red corresponds to the highest activation, i.e. attention. Top rows show the original images, while the bottom rows show activation heat maps. **(A-C)** correspond to the deformable sRBC class, while **(D-F)** correspond to the non-deformable sRBC class. For each panel consisting of cell images and class activation maps, the first column represent the original cell image with no implemented data augmentation. The next column, however, is the same cell image with additional data augmentations like reflection and rotation. The last 2 columns for each panel contain single cell images intentionally modified to remove certain regions (black blocks) in order to confuse the network. The number in each panel is the probability assigned by the network of the cell being a deformable **(A-C)** or a non-deformable **(D-F)** sRBC. For the deformable sRBCs in **(A-C)**, the network still classifies accurately when part of the dimple is blocked, but the probability drops when the entire dimple is blocked. Hence for these types of cells the dimple is the key distinguishing feature. Analogously for the non-deformable sRBC cell in **(D-F)** the network needs to see at least one sharp endpoint or majority of the edge to classify reliably.

To assess which features the fine-tuned ResNet-50 network used to distinguish adhered deformable and non-deformable cells, we generated class activation maps (CAMs) [51, 52]. At the lower and middle portion of the Phase II architecture, which corresponds to the ResNet-50 backbone, the model contains mostly convolutional layers that effectively act as Gabor filters after training [42], capable of general feature detection. The top part of the network (see Fig B in S1 Text) then does a global average 2D pooling and softmax to output probability predictions for the three classes. To visualize which image features most contributed to the classification decision, the CAM procedure focuses on this top portion of the network. Let *f*_*ijk*_ be the feature map output from the ResNet-50 backbone, with dimensions 7 × 7 × 2048. The global average pooling result is then a length 2048 vector $v_{k}=\frac{1}{49}\sum_{i,j}f_{ijk}$ that is input into softmax as *w*_*mk*_*v*_*k*_ + *b*_*m*_, where *w*_*mk*_ is a 3 × 2048 matrix of weights, and *b*_*m*_ is a length 3 bias vector. To generate the CAM for the *m*th object class, we calculate $c_{ij}^{\left(m\right)}=\sum_{k}w_{mk}f_{ijk}$, which corresponds to a coarse-grained 7 × 7 heat map of the image, indicating which regions contributed most to the classification decision for the *m*th class. After resizing to 32 × 32 pixels and translating the CAM magnitudes to an RGB color map, the resulting CAMs are overlayed on examples of the original images in Fig 7. Intriguingly, the highlighted features were similar to those used by human experts in making classification decisions: the dimple in partially deformable sRBCs and the sharp endpoints/edges for nondeformable sRBCs. Additionally, we can test the importance of the features by modifying the images to block out certain regions. Blocking out the features highlighted by the CAMs (such as the dimples for the deformable sRBCs) reduces the confidence of the network in the class assignment (indicated by the probabilities in each panel). Overall, this analysis illustrates that our fine-tuned ResNet-50 model focuses on relevant physical features in carrying out classification. This also hints at the potential of the network to generalize on never-before-seen channel images by leveraging these general cellular features.

### 3.5 Processing pipeline: Manual vs. machine learning performance

**Fig 8:**
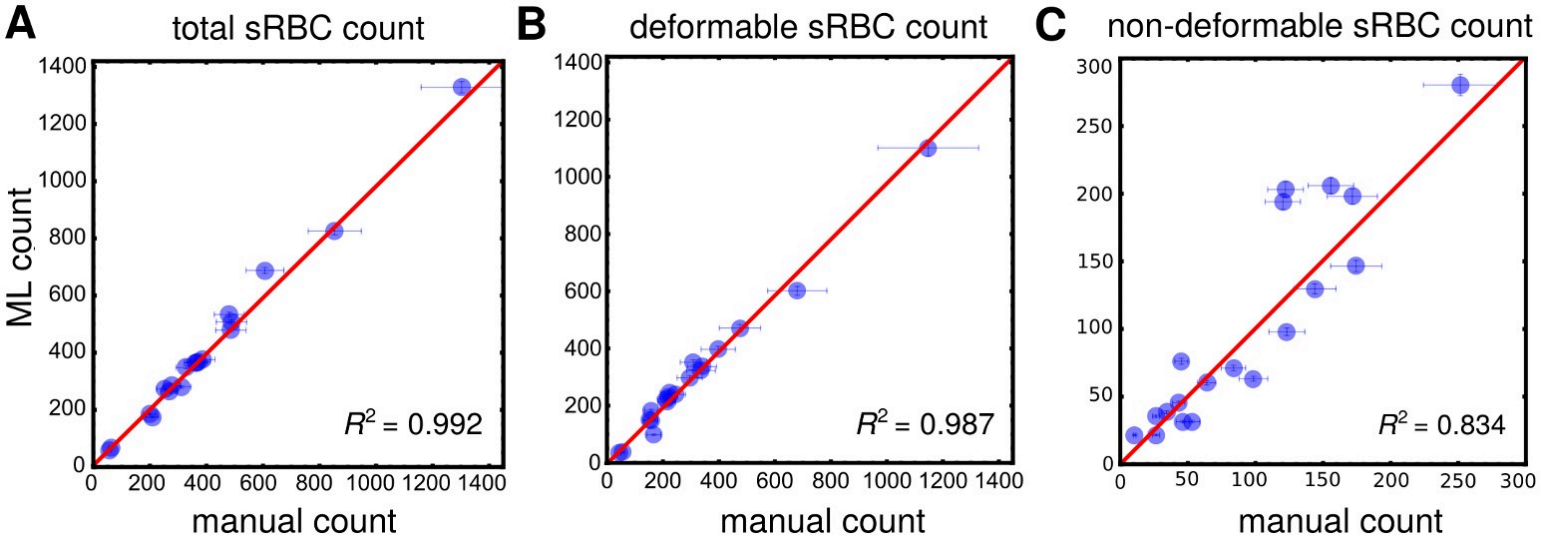
Manual vs machine learning (ML) performance. Results from pitting count estimates from 19 whole microchannel images processed through our automated two-part processing pipeline vs. manual characterization. Error bars along the manual axis are obtained from variance in repeated manual counts on a set of test images. The red line is the line of perfect agreement. Error bars on ML counts are estimated from the precision rates reached by our Phase II classifier network in predicting true positive outcomes in relevant categories on a validation set (see Fig 6). *R*^2^ statistic values, indicating goodness of agreement between manual and ML counts, are indicated in each graph. **A**: Results for total sRBC (deformable + nondeformable) cell counts. This plot is illustrative of the high degree of accuracy achieved by our ML in identifying sRBCs. **B** and **C**: Results for number of sRBCs in each channel image classified manually and by ML as deformable or non-deformable respectively. This measures the agreement reached in classification of the two morphological categories.

After both Phase I and II are complete in terms of training, we are ready for the final test of our processing pipeline, pitting the machine learning (ML) approach against 3 human experts in detecting, classifying, and counting adhered sRBCs for a set of 19 whole channel images displaying a wide variety of cells. All these 19 channels consisted of separate experiments from those used during training, and hence were never previously seen by the network. For this final test, we used a community of neural networks, called an ensemble model, in predicting segmentation masks and classifying/counting cells, improving performance and accuracy on test samples. In general, these ensemble models are known to perform better on a task than any single one model within the ensemble [53]. The Phase I and II ensemble models were assembled by collecting the 5 neural networks trained during *k*-fold cross-validation, and the final ensemble output was the just the average of the probabilities output from each of the individual networks. Importantly, this competition between human and ML will highlight the workflow’s true effectiveness for potentially replacing manual labor within biophysical studies of SCD. Results are illustrated in Fig 8. Error bars along the ML axis are obtained from the precision metrics of our classifier. Error bars on the manual axis are estimated from variance in repeated manual analyses on a set of whole channel images. Panel **A** shows results for total adhered sRBC cell count in each image, which can be taken as a proxy for overall object identification accuracy. We see how a very high degree of agreement is reached between our ML and human experts, with an estimated *R*^2^ statistic value of 0.992. Note how the manual error bars increase with sample size. This has serious implications for manual analyses of high cell count samples. A host of factors like longer duration of analysis time, mental fatigue of the experimentalist, etc. can affect these numbers. An ML-based automated classifier is immune to these human limitations. Panel **B** and **C** show comparison results for subcounts of deformable and non-deformable sRBCs in each sample image, indicative of classification accuracy. Excellent agreement is reached for deformable cells, with corresponding *R*^2^ ∼ 0.987. For non-deformable cells—a category significantly harder to identify because of the high degree of cross-correlating features with several objects in our “other” category—decent ML-manual agreement is still achieved, with *R*^2^ ∼ 0.834. In addition, our pipeline with a fine-tuned ResNet-50 model in Phase II outperforms the vanilla and Xception variants in all three categories in terms of the *R*^2^: total, deformable, and non-deformable counts (Table B in S1 Text). This gives us confidence in our chosen architecture. We note that our human experts showed significant variance among themselves in categorizing these kinds of images. The SCD pathogenetic pathway exhibits a continuum of physiological features, which we are trying to sort into discrete categories. Thus some level of disagreement is expected, particularly for images with borderline features between classes (e.g. Fig 1B(iii) and 1C(i)). The role of the ML then goes beyond simply matching human performance. It takes on the role of a standardized arbiter for these borderline images, and improves overall consistency in results compared to human performance, where random, spur-of-the-moment classification decisions would play a sizeable role.

## 4 Conclusion

We designed and tested a deep learning image analysis workflow for a microfluidic SCD adhesion assay that reduces the need for expert user input, enabling the processing of large amounts of image data with highly accurate results. For this study our target problem was identifying sRBCs in complex, whole channel bright field images using clinical whole blood samples, and distinguishing between their morphological subtypes (deformable and non-deformable). These subtypes are in turn strongly correlated with sRBC biomechanical properties, making the image analysis method a fast, high throughput proxy for the much more laborious cell-by-cell direct measurement of membrane deformability. We demonstrated that our network performed well in terms of accuracy when pitted against trained personnel, while improving analysis times by two orders of magnitude.

This proof-of-concept study focuses on sRBC deformable and non-deformable classification, but this is by no means the only feature worth exploring. We are working on generalizing our workflow to examine patient heterogeneities along more expanded metrics like white blood cell (WBC) content, WBC versus sRBC content, emergent sub-types and so on. Clinical heterogeneities among SCD-affected patients constitute a critical barrier to progress in treating the disease, and understanding them will be crucial in designing targeted patient-specific curative therapies. Increasing the frequency of therapeutic interventions, novel screening technologies, and better management of both acute short term and chronic SCD complications have gone a long away in increasing patient survival. The challenge lies in achieving targeted patient-specific administration of appropriate therapies, due to the wide heterogeneity among clinical sub-phenotypes. Developing tools for consistent and comprehensive monitoring of heterogeneities among patient groups is thus paramount. Emerging potentially curative therapies like allogenic hematopoietic stem cell transplantation (HSCT) and targeted gene therapy are also promising and becoming more widely available [24–26], but mostly in developed countries. These treatments need to be streamlined and standardized, requiring fast and affordable monitoring tools for assessment of efficacy checkpoints and endpoint outcomes along multiple relevant metrics. The workflow presented here, designed for integration with the SCD BioChip microfluidic assay, is a first step toward the ultimate goal of delivering such an ML-enabled SCD monitoring platform.

## Supporting information

S1 Text Supporting information. The supporting information contains figures and tables detailing performance comparisons of our approach with alternative Phase I and Phase II network architectures. **Fig A: Comparative performance of the Phase I network against another recent segmentation model, HR-net**. Training and validation history of performance metrics for the two networks, with our encoder-decoder in the top row, and HR-net in the bottom row. The solid curve corresponds to the 5-fold mean of each metric, while the same colored light band denotes the spread in the corresponding metric over these folds. Training history is shown in red and validation in blue (purple indicates overlap). To see the architecture details for both the encoder-decoder and HR-net, follow this link: https://github.com/hincz-lab/DeepLearning-SCDBiochip/blob/master/Demonstrate_architectures.ipynb. **Fig B: Phase II architecture**. A schematic outline of the architectures for our choice of Phase II network (ResNet-50) and two other networks used for performance comparison: a vanilla network and Xception. We appended a global average pooling layer along with a fully connected layer so that we can fine-tune the ImageNet pretrained models, e.g. ResNet-50 and Xception backbones, on our sickle red blood cell task. All of the tensor shapes shown in the cartoon illustration correspond to the input features, intermediate features, and output probability vector. **Fig C: Comparative performance of Phase II network against two other models—a vanilla network and Xception**. Training and validation history of performance metrics for the three networks are shown here. The solid curve corresponds to the mean training history over 5 folds, while the same colored light band denotes the spread in the corresponding metric over these folds. Training history is shown in red and validation in blue (purple indicates overlap). **Table A: Final metric values (averaged over 5 folds) reached by each Phase II network at the end of training**. This corresponds to the 30th, 50th and 30th epochs for ResNet-50, the vanilla network, and Xception respectively. Uncertainties indicate spread (standard deviation) around the mean of each metric over the 5 folds. The best achieved metric value over all networks is shown in bold, for both training and validation. While Xception does marginally better than ResNet-50 in training, it overfits more—validating our final choice of ResNet-50 for Phase II network based on overall performance. **Table B: Comparison of overall evaluation metrics for various pipeline configurations (Phase I + Phase II) on the sample set of 19 whole channel images**. *R*^2^ values are shown for the machine learning vs. manual count comparison in each case, similar to main text Fig 8. Table legend: ED: Encoder-decoder; CE Jaccard: Cross entropy Jaccard loss; All: total sRBC counts; Def: deformable sRBC counts; NDef: non-deformable sRBC counts; Proc. time: total processing time for all 19 channels.
(PDF)
