# Supplementary material for: Integrating deep learning with microfluidics for biophysical classification of sickle red blood cells adhered to laminin
Source: PLoS Comput Biol. 2021 Nov 29;17(11):e1008946. doi: 10.1371/journal.pcbi.1008946 (PMC8659663; doi:10.1371/journal.pcbi.1008946)
Supplement: S1 Text — The supporting information contains figures and tables detailing performance comparisons of our approach with alternative Phase I and Phase II network architectures. Fig A: Comparative performance of the Phase I network against another recent segmentation model, HR-net. Training and validation history of performance metrics for the two networks, with our encoder-decoder in the top row, and HR-net in the bottom row. The solid curve corresponds to the 5-fold mean of each metric, while the same colored light band denotes the spread in the corresponding metric over these folds. Training history is shown in red and validation in blue (purple indicates overlap). To see the architecture details for both the encoder-decoder and HR-net, follow this link: https://github.com/hincz-lab/DeepLearning-SCDBiochip/blob/master/Demonstrate_architectures.ipynb. Fig B: Phase II architecture. A schematic outline of the architectures for our choice of Phase II network (ResNet-50) and two other networks used for performance comparison: a vanilla network and Xception. We appended a global average pooling layer along with a fully connected layer so that we can fine-tune the ImageNet pretrained models, e.g. ResNet-50 and Xception backbones, on our sickle red blood cell task. All of the tensor shapes shown in the cartoon illustration correspond to the input features, intermediate features, and output probability vector. Fig C: Comparative performance of Phase II network against two other models—a vanilla network and Xception. Training and validation history of performance metrics for the three networks are shown here. The solid curve corresponds to the mean training history over 5 folds, while the same colored light band denotes the spread in the corresponding metric over these folds. Training history is shown in red and validation in blue (purple indicates overlap). Table A: Final metric values (averaged over 5 folds) reached by each Phase II network at the end of training. This corresponds t [file pcbi.1008946.s001.pdf]

Supporting Information:

Integrating deep learning with microfluidics for biophysical classification of sickle red blood cells adhered to laminin

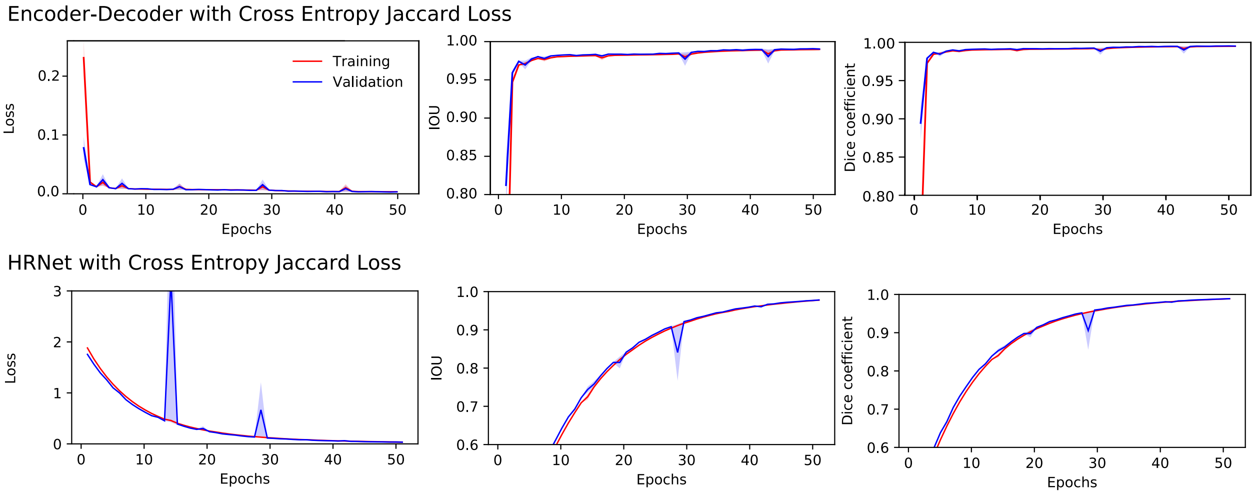

Fig A: **Comparative performance of the Phase I network against another recent segmentation model, HR-net**: Training and validation history of performance metrics for the two networks, with our encoder-decoder in the top row, and HR-net in the bottom row. The solid curve corresponds to the 5-fold mean of each metric, while the same colored light band denotes the spread in the corresponding metric over these folds. Training history is shown in red and validation in blue (purple indicates overlap). To see the architecture details for both the encoder-decoder and HR-net, follow this link: `https://github.com/hincz-lab/DeepLearning-SCDBiochip/blob/master/Demonstrate_architectures.ipynb`.

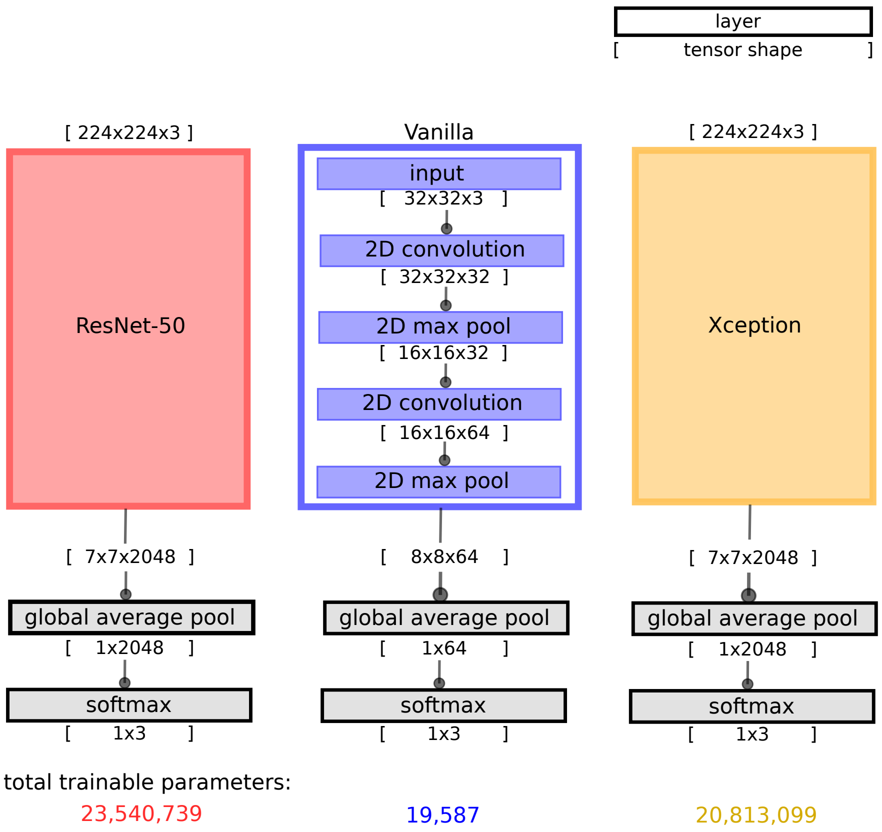

Fig B: **Phase II architecture**: A schematic outline of the architectures for our choice of Phase II network (ResNet-50) and two other networks used for performance comparison: a vanilla network and Xception. We appended a global average pooling layer along with a fully connected layer so that we can fine-tune the ImageNet pretrained models, e.g. ResNet-50 and Xception backbones, on our sickle red blood cell task. All of the tensor shapes shown in the cartoon illustration correspond to the input features, intermediate features, and output probability vector.

Network	ResNet-50		Vanilla		Xception	
Metric	Train	Val	Train	Val	Train	Val
Accuracy	0.981±0.001	**0.960±0.003**	0.800±0.002	0.795±0.008	**0.988±0.002**	0.949±0.010
Precision	0.982±0.001	**0.962±0.003**	0.830±0.001	0.827±0.010	**0.988±0.002**	0.950±0.010
Recall	0.981±0.001	**0.959±0.004**	0.763±0.002	0.760±0.005	**0.988±0.002**	0.949±0.010
F1 score	0.981±0.001	**0.960±0.003**	0.795±0.001	0.791±0.007	**0.988±0.002**	0.950±0.010

Table A: **Final metric values (averaged over 5 folds) reached by each Phase II network at the end of training.** This corresponds to the 30th, 50th and 30th epochs for ResNet-50, the vanilla network, and Xception respectively. Uncertainties indicate spread (standard deviation) around the mean of each metric over the 5 folds. The best achieved metric value over all networks is shown in bold, for both training and validation. While Xception does marginally better than ResNet-50 in training, it overfits more—validating our final choice of ResNet-50 for Phase II network based on overall performance.

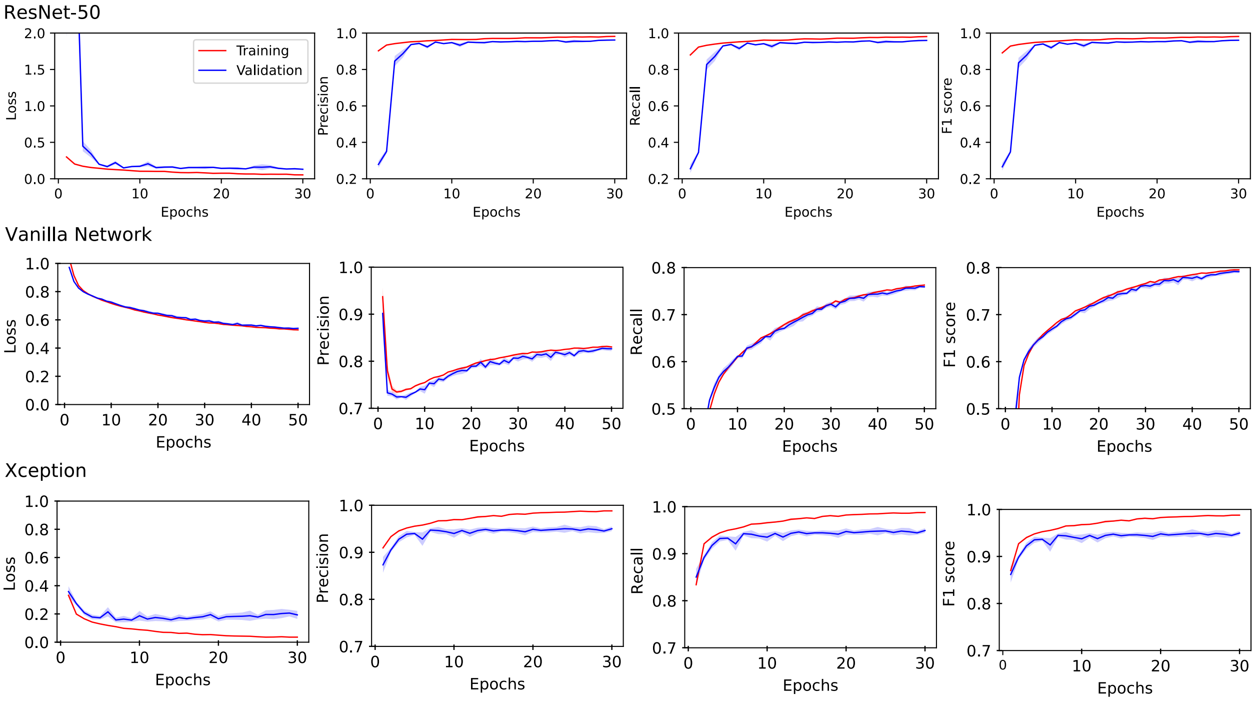

Fig C: **Comparative performance of Phase II network against two other models—a vanilla network and Xception**: Training and validation history of performance metrics for the three networks are shown here. The solid curve corresponds to the mean training history over 5 folds, while the same colored light band denotes the spread in the corresponding metric over these folds. Training history is shown in red and validation in blue (purple indicates overlap)

Pipeline Configuration			R^2		Proc.time
Phase I	Phase II	All	Def	NDef	(min)
ED CE Jaccard	ResNet-50	**0.9916**	**0.9875**	**0.8344**	22.91
ED CE Jaccard	Vanilla	0.9857	0.9632	0.7132	18.28
ED CE Jaccard	Xception	0.9896	0.9873	0.8317	21.64
HRNet CE Jaccard	ResNet-50	0.9814	0.9850	0.8038	45.39
HRNet CE Jaccard	Vanilla	0.9834	0.9680	0.7295	42.23
HRNet CE Jaccard	Xception	0.9798	0.9850	0.7935	45.94

Table B: **Comparison of overall evaluation metrics for various pipeline configurations (Phase I + Phase II) on the sample set of 19 whole channel images.** R^2 values are shown for the machine learning vs. manual count comparison in each case, similar to main text Fig 8. **Table legend:** ED : Encoder-decoder; CE Jaccard: Cross entropy Jaccard loss; All: total sRBC counts; Def: deformable sRBC counts; NDef: non-deformable sRBC counts; Proc. time: total processing time for all 19 channels.
